# Supplementary material for: ITPKC as a Prognostic and Predictive Biomarker of Neoadjuvant Chemotherapy for Triple Negative Breast Cancer
Source: Cancers (Basel). 2020 Sep 25;12(10):2758. doi: 10.3390/cancers12102758 (PMC7601042; doi:10.3390/cancers12102758)
Supplement: Supplementary file 1 [file cancers-12-02758-s001.pdf]

Supplementary Materials:

ITPKC as a Prognostic and Predictive Biomarker of Neoadjuvant Chemotherapy for Triple Negative Breast Cancer

Masanori Oshi, Stephanie Newman, Vijayashree Murthy, Yoshihisa Tokumaru, Li Yan, Ryusei Matsuyama, Itaru Endo and Kazuaki Takabe

Table 1. Clinical characteristics of low and high *ITPKC* expression in TNBC patients in TCGA cohort.

**Clinical variables**	***ITPKC*-Low** (***n*** **= 133)**	***ITPKC*-High** (***n*** **= 45)**	***p*-value**
Age at diagnosis			0.822
Median	48.0	51.0	
IQR	41-58	40-59	
AJCC T-category			0.409
T1	5 (3.8%)	4 (8.9%)	
T2	64 (48.1%)	21 (46.7%)	
T3	43 (32.3%)	11 (24.4%)	
T4	21 (15.8%)	9 (20.0%)	
AJCC N-category			0.432
N-	35 (26.3%)	9 (20.0%)	
N+	98 (73.7%)	36 (80.0%)	
Pathological Grade			0.586
G1	1 (0.8%)	0 (0.0%)	
G2	14 (10.5%)	7 (15.6%)	
G3	105 (78.9%)	36 (80.0%)	
Stage at diagnosis			0.281
I	2 (1.5%)	2 (4.4%)	
II	67 (50.4%)	18 (40.0%)	
III	64 (48.1%)	23 (51.1%)	

IV	0 (0.0%)	0 (0.0%)	

AJCC; American Joint Committee on Cancer, IQR; interquartile range
